# Neglected extensor apparatus injury in the proximal interphalangeal joint: A case report

**DOI:** 10.1097/MD.0000000000022083

**Published:** 2020-09-04

**Authors:** Young-Keun Lee, Jong-Hyun Ko

**Affiliations:** Department of Orthopedic Surgery, Research Institute of Clinical Medicine of Jeonbuk National University – Biomedical Research Institute of Jeonbuk National University Hospital, Jeonju, Jeonbuk, Republic of Korea.

**Keywords:** chronic rupture, extensor mechanism, finger, proximal interphalangeal joint

## Abstract

**Rationale:**

The extensor tendon of the proximal interphalangeal (PIP) joint is highly complex, and failure to ensure suitable balance during treatment following an injury is likely to produce poor outcomes. We have achieved good outcomes with the primary repair of neglected extensor tendon rupture in the PIP joint, and thus report the case along with a review of the relevant literature.

**Patients concern:**

A 40-year-old right-handed female who works at a meat shop visited our clinic due to pain and active limitation of the range of motion (ROM) of the PIP joint of her left long finger. She had previously experienced a cut on the dorsal aspect of the third PIP joint while cutting meat about a year earlier but did not receive any specific treatment for the injury.

**Diagnosis:**

The patient was diagnosed with complete rupture of the central slip and lateral band in the PIP joint after investigation.

**Intervention:**

We successfully debrided the ruptured tendon and performed extensor tendon repair using the modified Kessler technique and epitendinous cross-over repair technique.

**Outcome:**

At the 12-month follow-up, the patient was completely asymptomatic and had optimal PIP joint ROM (0°–90°) in her left long finger.

**Lessons:**

Although the treatment of an extensor injury of the PIP joint area is difficult, satisfactory outcomes can still be achieved, even in cases of injuries which are neglected for over a year, using a repair technique that can properly balance the length and tension between the central slip and lateral bands with the selection of appropriate postoperative treatment strategies.

## 1 Introduction

Unlike the flexor tendons, the extensor tendons in the fingers are anatomically thin and flat and are vulnerable to bone and joint injuries due to little protection provided by soft tissues. Moreover, because the structure between the intrinsic and extrinsic tendons is complex, treatment tends to be more difficult in this area compared to other areas.^[1]^ In particular, the extensor apparatus in the proximal interphalangeal (PIP) joint is the site where the central slip and two lateral bands split. Thus, properly balancing the length and tension between the central slip and lateral bands during reconstruction is very important; however, it is not easy.^[2]^ Moreover, because this area has a higher probability of extension lag and adhesion after tendon repair than in other areas, early joint motion using a dynamic splint has been attempted postoperatively to prevent such occurrences.^[3,4]^ The authors of the present study achieved satisfactory outcome through primary repair in a patient with neglected extensor apparatus injury in the PIP joint area. Accordingly, this case is reported here together with a literature review.

### 1.1 Consent

The patient signed informed consent for the publication of this case report and any accompanying images. Ethical approval of this study was waived by the ethics committee of Jeonbuk National University Hospital because it was a case report and there were fewer than 3 patients.

## 2 Case presentation

**Figure 1:**
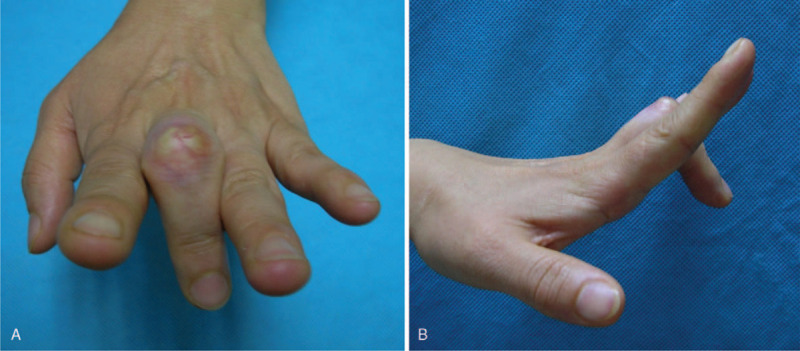
(A and B) Initial image after active extension of the proximal interphalangeal (PIP) joints of the left long finger shows an absence of extension at the PIP joints.

**Figure 2:**
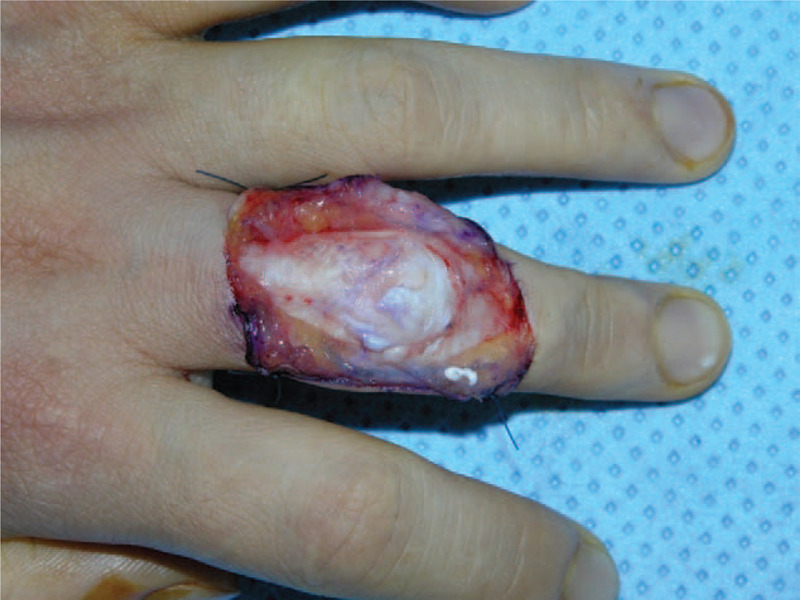
Intraoperative photograph shows complete rupture of the central slip and ulnar lateral band. The remnant of the distal stump of the central slip was about 1 cm.

**Figure 3:**
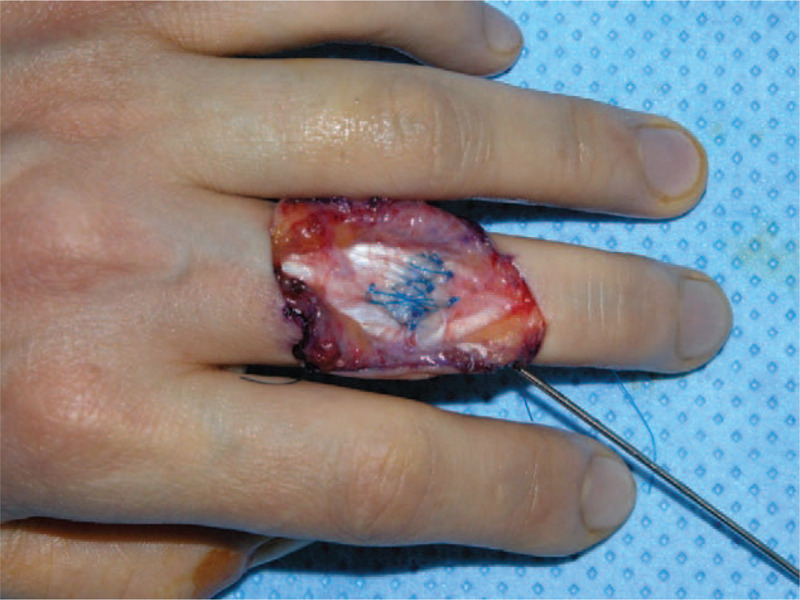
Intraoperative photographs showing the central slip repair with the modified Kessler and epitendinous cross-over methods after the denatured soft tissue was removed. The proximal interphalangeal joint was fixed with a transarticular K-wire. (Central slip repair was performed by debridement of the stump area, following by tenolysis after fixing the extended PIP joint with 1.0 mm K-wire; advancing the proximal stump; and repairing the area with modified Kessler technique and epitendinous cross-over repair.)

**Figure 4:**
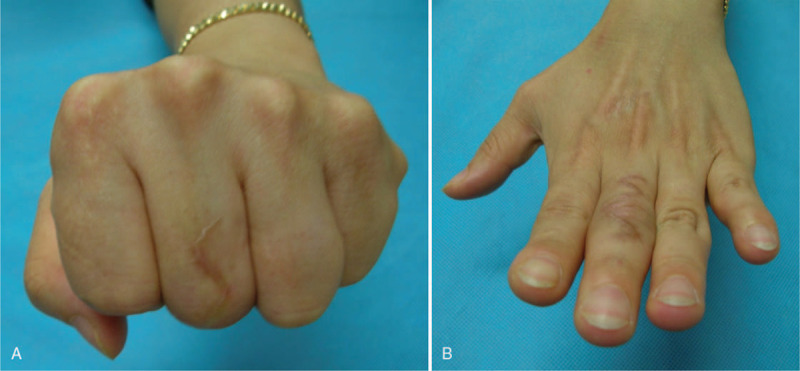
(A and B) Photographs obtained 12 months after the operation showing excellent proximal interphalangeal joint range of motion.

A 40-year-old right-handed female who works at a meat shop visited our clinic due to pain and active limitation of the range of motion (ROM) of the PIP joint of her left long finger (Fig. 1A and B). She had previously experienced a cut on the dorsal aspect of the third PIP joint while cutting meat about a year earlier but did not receive any specific treatment for the injury. A physical examination revealed a wound scar and thinning of the skin. Passive ROM in the PIP and distal interphalangeal (DIP) joints was normal, and extension could be maintained when the PIP joint was passively extended. With the patient under regional anesthesia, the extensor tendon was explored through a dorsal zig–zag incision. Surgical findings showed complete rupture of the central slip and lateral bands in the PIP joint area, with ∼1 cm of the distal stump of the central slip remaining (Fig. 2). Debridement was performed on the stump, followed by tenolysis after fixing the extended PIP joint using a 1.0-mm Kirschner (K)-wire, advancing the distal stump, and repairing the area with 4-0 prolene sutures (PROLENE 4-0 Suture, ETHICON, Cincinnati, OH) using a modified Kessler technique and epitendinous cross-over repair (Fig. 3). At postoperative 4 weeks, the K-wire was removed and a dynamic splint was applied to initiate active joint motion. At the 12-month follow-up, the patient was completely asymptomatic and had excellent PIP joint ROM (0°–90°) in her left long finger (Fig. 4A and B).

## 3 Discussion

The extensor apparatus in the PIP joint, involving the central slip, lateral bands, and intrinsic tendon, is anatomically complex and dynamic.^[2]^ Although it is rare for all these structures to be injured by a laceration in the dorsal aspect of a finger, extensor injury in this area can be repaired easily by a simple interrupted repair or the Silfverskiöld cross-stitch technique.^[5]^ However, because the extensor tendon is located on the extra synovium and close to the PIP bone, it represents one of the parts with poorest postoperative functional recovery in the extensor tendon area. In particular, if a laceration of the central slip is left untreated, a buttonhole deformity may occur, and because this is one of the deformities that is difficult to reconstruct when it occurs in the hand, the prevention of such deformity through aggressive treatment is important.

In the present case, we treated a patient with a buttonhole deformity for a central slip injury that was neglected for over a year. Reconstruction methods for a chronic buttonhole deformity can be divided into three major types—tenotomy, tendon grafting, and tendon relocation—which may be applied according to the stage of chronic buttonhole deformity.^[5]^ Curtis et al^[6]^ reported that it is useful to treat chronic buttonhole deformity using a stepwise approach with procedures performed in the order, extensor tenolysis, transverse carpal ligament dissection, when necessary, lateral ligament lengthening from the middle phalanx, and central slip repair.

Central slip repair in the PIP joint include various methods. Woo et al^[7]^ reported that in a biomechanical comparison according to four types of repair techniques in zone IV of the extensor tendon, the modified Becker and modified Kessler techniques had stronger maximum loading that other techniques, and tendon shortening was less severe. In the present case, since there was enough distal stump of the central slip remaining for primary repair, the authors performed tenolysis, followed by the modified Kessler technique on the central slip and lateral bands, and epitendinous cross stitching. The PIP joint was fixed with a transarticular K-wire while it was extended.

With respect to treatment subsequent to the central slip repair, Doyle^[8]^ stated that active joint motion of the PIP joint should be initiated after removing the K-wire after 4 weeks, and to keep the PIP joint extended for 2 to 4 months with a splint. In a report on treatment outcomes using an active splint on an actual extensor injury in zone III of the extensor tendon, Saldana et al^[4]^ recommended that, instead of early motion, PIP joint motion should not be allowed for at least 1 month after the extensor repair. Here, we removed the K-wire after 4 weeks and applied a dynamic splint for initiating active joint motion.

## 4 Conclusion

Although the treatment of an extensor injury in the PIP joint area is difficult, satisfactory outcomes can still be achieved, even in cases of injuries neglected for over a year, using a repair technique that can properly balance the length and tension between the central slip and lateral bands and selection of appropriate postoperative treatment strategies.

## Author contributions

**Writing – original draft:** Young keun Lee.

**Writing – review & editing:** Young Keun Lee, Jong Hyun Ko.
